# Return to Sport Following Anterior Cruciate Ligament Reconstruction: A Cross-Sectional Study in Saudi Arabia

**DOI:** 10.7759/cureus.82965

**Published:** 2025-04-25

**Authors:** Salahulddin Abuljadail, Azzam O Albotuaiba, Ali S Almarri, Abdullah K Alhamam, Abdulmohsen K Almulhim

**Affiliations:** 1 Department of Orthopaedic Surgery, King Faisal University, Al Ahsa, SAU; 2 College of Medicine, King Faisal University, Al Ahsa, SAU

**Keywords:** aclr, anterior cruciate ligament, anterior cruciate ligament (acl), anterior cruciate ligament reconstruction (aclr), biopsychosocial model, ligament repair, pre-injury activity, return to sport

## Abstract

Introduction

The anterior cruciate ligament (ACL) is essential for knee stability, and its injury significantly impacts athletic performance. Anterior cruciate ligament reconstruction (ACLR) is the standard treatment for active individuals, yet many fail to return to sport (RTS) due to factors like pain and kinesiophobia. This study explores these barriers in the Saudi Arabian population.

Methodology

This is a cross-sectional study using an online questionnaire targeting ACLR patients across five Saudi hospitals between 2017 and 2023. Tools included are the International Knee Documentation Committee’s (IKDC) scale and the Tampa Scale for Kinesiophobia (TSK-11) score. Data was analyzed using IBM SPSS Statistics software, version 29.0.0 (IBM Corp., Armonk, NY).

Results

Our study included 84 participants undergoing ACL reconstruction. Most were aged between 18 and 25 years (n=35, 41.7%), male (n=75, 89.3%), and employed (n=65, 77.4%). Only 44.0% (n=37) returned to their pre-injury level of sport. Moderate fear of re-injury was common (n=67, 79.8%), and fear significantly correlated with lower IKDC scores (r = -0.410, p < 0.001). Participants with minimal fear had the highest IKDC score (81.01 ± 9.47, p < 0.001). Functional limitation was significantly associated with RTS (p < 0.001); none with moderate/severe limitations returned. Lower pain scores (1.77 vs. 4.35, p = 0.005), lower TSK-11 scores (15.77 vs. 22.90, p < 0.001), and higher knee function (81.01 vs. 63.02, p < 0.001) were significantly linked to RTS. Height > 180 cm was also significantly associated with RTS (p = 0.011).

Conclusion

Our findings show that returning to sport after ACLR is strongly influenced by functional ability, pain severity, and fear of re-injury. Psychological readiness, particularly kinesiophobia, plays a critical role. Addressing both physical limitations and mental barriers is essential to improve RTS outcomes in post-ACLR patients.

## Introduction

The anterior cruciate ligament (ACL) is an intracapsular ligament that runs from the posteromedial aspect of the lateral epicondyle to the intercondylar eminence of the tibia [1]. It plays a great role in both knee stabilization and function. The ACL prevents anterior translation of the tibia as well as limits the excessive valgus rotation of the knee joint [2]. The ACL is considered the primary stabilizer of the knee joints. Thus, the ACL's role in knee stability is irreplaceable. Its rupture in active athletic individuals leads to grave consequences. ACL rupture greatly hinders athletic performance and increases the risk of early degenerative changes [3]. It is one of the most common injuries of the knee joint, with an incidence of 85 individuals per 100,000 of the population. ACL injuries could occur through contact or noncontact mechanisms. Around 75% of the cases are acquired through minimal or noncontact mechanisms [4]. The most common noncontact mechanism of injury is rapid alternating movement while bearing weight on a slightly flexed or internally rotated knee [5]. Abrupt deceleration movement to change directions has also been seen to cause ACL [6]. The management can be surgical or conservative. The decision is based on multiple factors, including the level of activity, the nature of the occupation, and the ability to adhere to rehabilitation protocols. The conservative non-surgical approach is reserved for old (aged > 54 years) sedentary patients with minimal risk of re-injury. Young, active individuals with a higher risk of re-injuries are candidates for anterior cruciate ligament reconstruction (ACLR) [7]. Thus, it is considered the gold standard to treat previously active patients [8]. During ACLR, the torn ACL is removed, and either an autograft or allograft is used to replace the deficient ACL. Both the patellar tendon (PT) and the hamstring tendon (HT) are commonly used, with no difference between them in terms of functional outcome and physical activity [9]. Allografts can also be used but have a higher rate of failure and infection [10, 11]. Several factors affect the success of ACLR surgery, such as graft choice, timing of surgery, and pre- and post-reconstruction rehabilitation [8]. Early repair is associated with an increased incidence of arthrofibrosis, leading to joint stiffness and loss of 5° of full knee extension. This devastating complication may require arthroscopic lysis of adhesions (LOA) [12]. Delayed repair increases muscle weakness and potentially leads to further injuries [8]. Conservative management of low-activity patients involves rehabilitation, bracing of the deficient knee, and activity modification. In both approaches, well-structured rehabilitation programs are essential to improve patients’ outcomes [7].

The goal of ACLR surgery is to achieve a pre-injury level of activity, ensuring that patients return to sport (RTS) or a pre-injury level of physical activity within nine months of surgery [13]. Although 90% of patients have successful outcomes after ACLR, only 63% return to pre-injury sports. Even after clearance is given after rehabilitation, many don’t return to pre-injury activities due to factors not related to knee function [14]. The presence of such a discrepancy raises the question of psychological involvement. Reasons for non-returning include choice-related and persistent residual symptoms. Choice-related factors include fear of re-injury (kinesiophobia) and lack of motivation. Kinesiophobia has been defined as “an irrational and debilitating fear of physical movement resulting from a feeling of vulnerability to painful injury or re-injury [15]. Patients are afraid to have to experience the pain again. Consequently, they avoid high-intensity physical activity or sport. Patients may also lose their position on the sports team and lose motivation with it. Other non-psychological factors, such as residual symptoms, could contribute to the issue as well. The most common symptoms cited by patients include pain and swelling. Stiffness, instability, or weakness are less commonly cited as a reason for not returning [16]. We hypothesize that factors such as kinesiophobia and pain significantly affect the rates of RTS. Addressing these factors could greatly improve the end results. 

To the best of our knowledge, only one study has been conducted in Saudi Arabia examining factors affecting RTS. The study involved a small sample size (93 participants); 553 were previously contacted to participate. Only 21% responded. The participants were predominantly males (98.9%). Consequently, it is not appropriate to generalize their findings in societies with higher female participation in sport [17]. Furthermore, the study was conducted only in one tertiary center. Factors specific to this center could skew the results. The aim of our study is to assess the various factors affecting RTS. The objective of this study is to examine the significant relationships between fear of re-injury, pain levels, and rates of return to sport or pre-injury levels of physical activity. We aim to add to the scarce body of literature in this field in Saudi Arabia.

## Materials and methods

Research design

This was a cross-sectional study. Data were collected through an online questionnaire using Google Forms (Google LLC, Mountain View, CA). A retrospective review of patients who underwent ACLR in the Ministry of National Guard Health Affairs from January 2017 to June 2023. Five hospitals are spread from east to west throughout Saudi Arabia, located in Al Ahsa, Dammam, Riyadh, Madinah, and Jeddah. The total bed capacity is 3,737 beds and provides all levels of care to National Guard personnel, their dependents, and the citizens. The largest center is in Riyadh with 2,151 beds. All centers are linked to one electronic medical record system (BestCare, ezCaretech; Seoul, South Korea). The patients were contacted to participate in the study.

Ethical approval was obtained from the King Abdullah International Medical Research Center (KAIMARC), Riyadh, Saudi Arabia. Informed consent was obtained from all participants prior to their participation. Research participants were allowed to quit the survey at any time they desired. This study was anonymous, and the information was confidential.

Study sample

The total sample size of the study participants was calculated by the Raosoft online calculator (Raosoft Inc., Seattle, WA). The desired sample size was 383 individuals. The response distribution was assumed to be 50% with a confidence interval of 95% and a 5% margin of error. But the original response rate is 21.9%, with 84 participants completing the survey. The questionnaire was circulated online, and participants were encouraged to share the questionnaire with acquaintances who have undergone ACLR.

Inclusion and exclusion criteria

The current study included adult (18 years and older) male and female patients who were living in Saudi Arabia and who were diagnosed with ACL injury and underwent ACLR. Participants younger than 18 years, not living in Saudi Arabia, who were not diagnosed with ACL injury or did not have ACLR were excluded. Participants with previous knee surgery, neurovascular injury, fractures around the knee joint, or concomitant meniscal or other ligamentous injuries were also excluded from the study.

Measures

In order to achieve the goals of the present study, different measures were used. Tools included were the International Knee Documentation Committee’s (IKDC) scale and Tampa Scale for Kinesiophobia (TSK-11) (Appendix A). The demographic questionnaire prepared by the researcher was also included in the study (Appendix B).

Demographic Questionnaire

The information about the demographic profile of the participants was collected with the help of questions related to their age, sex, marital status, and occupation. Data on BMI were also collected. Patients were asked whether they suffered from osteoarthritis or not. Information related to the injury, including the site of the injury, the time between injury and surgery, time since surgery, and concomitant knee injury (including meniscal and ligamentous injury), was collected.

IKDC Form

An Arabic validated version of IKDC was used to assess postoperative knee function as a measure of treatment effectiveness [18] (Appendix C). IKDC is a subjective measure of knee function after ACLR. The IKDC has 10 questions. The score ranges from 0 to 100, with the highest score representing better knee function [19].

TSK-11

TSK-11, a shortened version of TSK, was originally designed to assess chronic pain syndromes of the musculoskeletal system. Following a musculoskeletal injury, fear of experiencing pain is the primary factor in determining recovery. Avoidance and escape behavior delay recovery [20]. TSK-11 has been proven to be a valid and reliable instrument for the assessment of kinesiophobia and fear of reinjury after ACLR. TSK-11 contains 11 items, with the lowest score indicating no or little kinesiophobia and fear of re-injury. The highest score is 44 and indicates extreme kinesiophobia and fear of re-injury [21].

Statistical analysis

A comprehensive statistical analysis was conducted on the dataset, encompassing both descriptive and inferential methodologies. A descriptive analysis is conducted to summarize the demographic characteristics of the participants, which include age, gender, and other features. Moreover, Fisher’s exact test was used to assess the association between categorical variables. An independent t-test was used to determine the association between continuous variables. Subsequently, Pearson’s correlation was used to find the correlation between scores. All statistical analyses were executed using IBM SPSS Statistics software, version 29.0.0 (IBM Corp., Armonk, NY).

## Results

Our study included 84 participants for RTS following ACLR (Table 1). Notably, the majority were aged between 18 and 25 years (n=35, 41.7%), male (n=75, 89.3%), and married (n=52, 61.9%). Most participants were employed (n=65, 77.4%), with the predominant height range being 166-180 cm (n=60, 71.4%) and weight between 81-95 kg (n=27, 32.1%). Arthritis was reported by 26 participants (31.0%). Injuries were more common on the right side (n=33, 39.3%), followed by the left (n=25, 29.8%). Notably, 30 participants (35.7%) underwent ACLR more than 12 months after injury. Most had no concurrent ligament or knee fractures (n=65, 77.4%).

**Table 1:** Sociodemographic and injury-related parameters of participants (n=84) ACLR: anterior cruciate ligament reconstruction

Parameters with responses		Frequency N (%)
Age	18-25 years	35 (41.7%)
	26-40 years	22 (26.2%)
	>40 years	27 (32.1%)
Gender	Female	9 (10.7%)
	Male	75 (89.3%)
Married	No	32 (38.1%)
	Yes	52 (61.9%)
Employee	No	19 (22.6%)
	Yes	65 (77.4%)
Height (cm)	<155 cm	1 (1.2%)
	155-165 cm	18 (21.4%)
	166-180 cm	60 (71.4%)
	>180 cm	5 (6.0%)
Weight	50-65 kg	17 (20.2%)
	66-80 kg	18 (21.4%)
	81-95 kg	27 (32.1%)
	>95 kg	20 (23.8%)
Arthritis	No	57 (67.9%)
	Yes	26 (31.0%)
Where is the Injury?	Right	33 (39.3%)
	Left	25 (29.8%)
	Both	4 (4.8%)
Time from injury to surgery (ACLR)?	<1 month	9 (10.7%)
	1-6 months	9 (10.7%)
	6-12 months	12 (14.3%)
	>12 months	30 (35.7%)
Any other ligament injuries/fractures in the knee?	No	65 (77.4%)
	Yes	15 (17.9%)

Table 2 shows participants' responses regarding fear of movement due to anticipated pain or reinjury (n=84). A substantial proportion agreed or strongly agreed that they were afraid of reinjury with exercise (n=37, 44.0%; n=8, 9.5%). The belief that pain would worsen if they tried to overcome it was common (n=49, 58.3%), with no one strongly agreeing. Notably, 52.4% (n=44) agreed and 4.8% (n=4) strongly agreed that pushing through pain increases discomfort. Almost half (n=40, 47.6%) felt it was unsafe to be active, and the same proportion felt they couldn’t do what others could without risking injury. A majority (n=46, 54.8%) agreed that no one should exercise in pain. Concerns of permanent bodily risk post injury were noted in 41.7% (n=35), highlighting the psychological barrier to return to sport driven by perceived vulnerability and danger.

**Table 2:** Assessment of fear of movement due to anticipated pain or injury which impacts participants’ decision to return to sport (n=84)

Parameters with responses	Strongly disagree (N (%))	Disagree (N (%))	Agree (N (%))	Strongly agree (N (%))
I am afraid that I might injure myself if I exercise.	13 (15.5%)	26 (31.0%)	37 (44.0%)	8 (9.5%)
If I try to overcome it, my pain would increase.	7 (8.3%)	28 (33.3%)	49 (58.3%)	-
If I try to overcome it, my pain would increase.	2 (2.4%)	34 (40.5%)	44 (52.4%)	4 (4.8%)
It is really not safe for a person with a condition like mine to be physically active.	9 (10.7%)	27 (32.1%)	40 (47.6%)	6 (7.1%)
I can't do all the things that normal people do because it is too easy for me to get injured.	6 (7.1%)	31 (36.9%)	40 (47.6%)	7 (8.3%)
No one should have to exercise when he/she is in pain.	12 (14.3%)	26 (31.0%)	40 (47.6%)	6 (7.1%)
My body is telling me I have something dangerously wrong.	20 (23.8%)	40 (47.6%)	17 (20.2%)	7 (8.3%)
My accident has put my body at risk for the rest of my life.	15 (17.9%)	34 (40.5%)	26 (31.0%)	9 (10.7%)
I wouldn't have this much pain if there weren't something potentially dangerous going on in my body.	15 (17.9%)	38 (45.2%)	27 (32.1%)	4 (4.8%)

**Figure 1:**
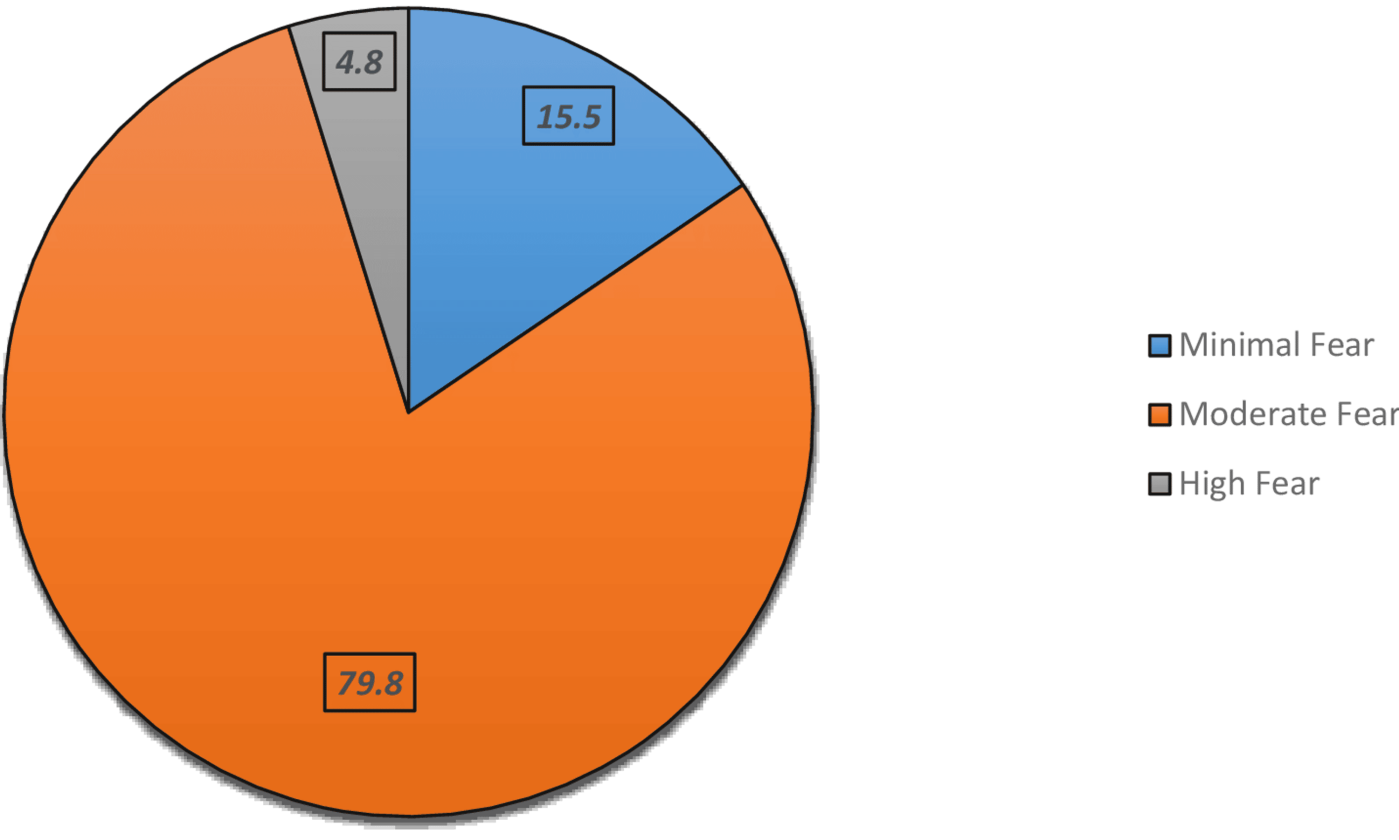
Overall level of fear, which impacts participants’ decision to return to sport based on the TSK-11 scale TSK-11: Tampa Scale for Kinesiophobia

Figure 1 shows the overall distribution of fear levels influencing participants’ decisions to RTS, based on the TSK-11 scale. A significant majority of participants (n=67, 79.8%) experienced moderate fear, indicating widespread psychological hesitancy towards re-engaging in physical activity post injury. Minimal fear was reported by only 15.5% (n=13) of participants, representing those with greater confidence in returning to sport. Notably, high fear was present in just 4.8% (n=4), though this small group likely faces the most significant psychological barriers.

Table 3 shows knee symptoms, activity limitations, and functional outcomes among 84 participants post injury. While 25.0% (n=21) could perform very strenuous activities, 29.8% (n=25) were restricted to simple tasks. Pain levels averaged 4.0 (SD 3.1), with similar scores for severity. Over half (n=46, 54.8%) had no swelling, but 33.3% (n=28) reported moderate to very severe swelling. Regarding stiffness, 57.1% (n=48) reported none, yet 41.7% (n=35) experienced persistent stiffness. Function without swelling or stiffness remained limited for many, though 22.6% (n=19) could still perform strenuous activity despite swelling or 17.9% (n=15) despite stiffness. Sports participation was affected: only 25.9% (n=21) reported engaging in very strenuous sports, while 30.9% (n=25) could only do basic activities. Functional impairments were most notable during squatting (36.6% very difficult), sitting with bent knees (29.3%), and kneeling (15.9%). High-impact tasks like jumping onto the injured leg (17.1% unable) and holding/moving quickly (21.5% difficulty) were also commonly impaired. Conversely, getting up from a chair showed better outcomes (51.2% no difficulty). The mean knee function score declined from 5.3 (SD 4.2) pre-injury to 4.5 (SD 3.2) post injury, indicating a noticeable functional decline.

**Table 3:** Assessment of knee symptoms, sports activity, and functional outcome of knee after injury (n=84)

Parameters with responses		Frequency N (%)
Symptoms		
Highest activity without knee pain	Can't play sports (pain)	5 (6.0%)
	Simple activities (household/walking)	25 (29.8%)
	Moderate (running/jogging)	21 (25.0%)
	Strenuous (tennis/skiing)	9 (10.7%)
	Very strenuous (football/cycling)	21 (25.0%)
During the past four weeks or since the injury, how much pain have you felt? (0-10 scale)	Mean (SD)	4 (3.1)
How severe is the pain? (0-10 scale)	Mean (SD)	4 (3)
Stiff or swollen knee (past four weeks)	No	46 (54.8%)
	Mild	10 (11.9%)
	Moderate	14 (16.7%)
	Severe	8 (9.5%)
	Very severe	6 (7.1%)
Highest activity without swelling	Unable to perform (knee dislocation)	4 (4.8%)
	Simple activities (household/walking)	26 (31.0%)
	Moderate (running/jogging/)	19 (22.6%)
	Strenuous (tennis/skiing)	16 (19.0%)
	Very strenuous (football/cycling)	19 (22.6%)
Stiffness since Injury	Never	48 (57.1%)
	Yes (constant)	35 (41.7%)
Highest activity without stiffness	Unable to perform (knee dislocation)	1 (1.2%)
	Simple activities (household/walking)	29 (34.5%)
	Moderate (running/jogging/)	23 (27.4%)
	Strenuous (tennis/skiing)	14 (16.7%)
	Very strenuous (football/cycling)	15 (17.9%)
Sports activity		
What is the highest level of activity you can participate in on a regular basis?	Can't play sports (pain/ knee dislocation)	5 (6.2%)
	Simple activities (household/walking)	25 (30.9%)
	Moderate (running/jogging)	21 (25.9%)
	Strenuous (tennis/skiing)	9 (11.1%)
	Very strenuous (football/cycling)	21 (25.9%)
Does your knee affect your ability to perform the following functions		
Climbing stairs	I can't	2 (2.4%)
	Very difficult	3 (3.7%)
	A little difficult	46 (56.1%)
	No difficulty	31 (37.8%)
Going down the stairs	I can't	2 (2.4%)
	Very difficult	5 (6.1%)
	A little difficult	35 (42.7%)
	No difficulty	40 (48.8%)
Kneeling	I can't	3 (3.7%)
	Very difficult	13 (15.9%)
	A little difficult	32 (39.0%)
	No difficulty	34 (41.5%)
Squatting	I can't	5 (6.1%)
	Very difficult	30 (36.6%)
	A little difficult	27 (32.9%)
	No difficulty	20 (24.4%)
Sitting with bent knees	I can't	6 (7.3%)
	Very difficult	24 (29.3%)
	A little difficult	32 (39.0%)
	No difficulty	20 (24.4%)
Getting up from the chair	I can't	6 (7.3%)
	Very difficult	4 (4.9%)
	A little difficult	30 (36.6%)
	No difficulty	42 (51.2%)
Jump back down onto your injured leg	I can't	14 (17.1%)
	Very difficult	12 (14.6%)
	A little difficult	30 (36.6%)
	No difficulty	26 (31.7%)
Hold and then move quickly	I can't	3 (3.8%)
	Very difficult	14 (17.7%)
	A little difficult	39 (49.4%)
	No difficulty	23 (29.1%)
Function (0=worst function, 10=good function)		
Function prior to your knee injury (0-10 scale)	Mean (SD)	5.3 (4.2)
Current function of your knee injury (0-10 scale)	Mean (SD)	4.5 (3.2)

**Figure 2:**
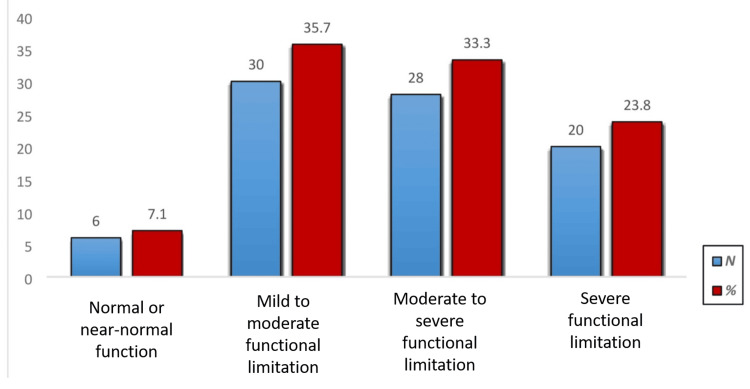
Overall functional capacity of knee of participants based on the IKDC scale IKDC: International Knee Documentation Committee

Figure 2 shows the distribution of knee functional capacity among participants based on the IKDC scale. Only a small portion of the cohort (n=6, 7.1%) achieved normal or near-normal knee function following injury. The majority fell into the categories of mild to moderate limitation (n=30, 35.7%) and moderate to severe limitation (n=28, 33.3%), indicating a substantial reduction in knee performance. Additionally, 20 participants (23.8%) reported severe limitations or dysfunction, reflecting significant impairment. These findings underscore that more than 92% of individuals experienced some degree of functional compromise.

Table 4 shows the relationship between fear of re-injury (TSK-11 scale) and knee functional ability (IKDC score) among participants (n=84). Participants with minimal fear had the highest mean IKDC score of 81.01 ± 9.47, indicating near-normal knee function. In contrast, those with moderate fear showed a reduced mean IKDC score of 63.85 ± 17.78, while high fear was associated with the lowest mean score of 49.05 ± 4.77, suggesting significant functional limitation. The difference across groups was statistically significant (p < 0.001, ANOVA), implying that poorer knee function is associated with increased fear of re-injury. Furthermore, a negative correlation was found between TSK-11 and IKDC scores (r = -0.410, p < 0.001, Spearman), indicating that knee function decreases due to fear of movement/re-injury related to sports.

**Table 4:** Assessment of relationship and correlation between fear of re-injury and return to sport or pre-injury level of physical activity (n=84) TSK-11: Tampa Scale for Kinesiophobia; IDKC: International Knee Documentation Committee

Level of fear in returning to sports based on the TSK-11 scale	Knee functional ability based on the IKDC score		Sig. value
	Mean	Sd	
Minimal fear	81.01	9.47	<0.001^a^
Moderate fear	63.85	17.78	
High fear	49.05	4.77	
Total	65.80	17.83	
Correlation between TSK-11 scale and IKDC score (r)	-0.410		<0.001^ b^

Table 5 shows factors associated with return to sport at pre-injury levels among 84 participants. Overall, 44.04% (n=37) returned to their previous physical activity. While age, gender, marital status, and employment did not show statistically significant associations with RTS, trends suggest that younger individuals (18-40 years) and males had higher RTS rates. Height showed a significant association (p = 0.011), with taller participants (>180 cm) returning more often. Interestingly, none of the participants who were shorter than 155 cm returned to sport. Although not statistically significant, left-sided injuries were less likely to result in RTS (20.0%) compared to right-sided (45.5%) or bilateral injuries (50.0%). Time from injury to surgery showed no clear pattern, but early surgery (<1 month) had the highest RTS rate (55.6%). A highly significant association was found between functional limitations and RTS (p <0.001). All participants with normal or mild-to-moderate functional limitations returned to sport, while none with moderate-to-severe limitations did. Lower pain scores (mean 1.77 vs. 4.35, p=0.005), less severe pain (p=0.016), lower TSK scores (mean 15.77 vs. 22.90, p<0.001), and higher IKDC scores (mean 81.01 vs. 63.02, p<0.001) were significantly associated with successful RTS.

**Table 5:** Association of different factors with the return to sport with pre-injury level of physical activity TSK-11: Tampa Scale for Kinesiophobia; IDKC: International Knee Documentation Committee

Parameters with responses		Return to sports		Sig. values
		No N (%)	Yes N (%)	
Age	18-25 years	18 (51.4%)	17 (48.6%)	0.112^ a^
	26-40 years	11 (50.0%)	11 (50.0%)	
	>40 years	18 (66.7%)	9 (33.3%)	
Gender	Female	5 (55.6%)	4 (44.4%)	0.343^ a^
	Male	42 (56.0%)	33 (44.0%)	
Married	No	17 (53.1%)	15 (46.9%)	0.227^ a^
	Yes	30 (57.7%)	22 (42.3%)	
Employment	Not employed	12 (63.2%)	7 (36.8%)	0.723^ a^
	Employed	35 (53.8%)	30 (46.2%)	
Height (cm)	<155 cm	1 (100.0%)	0 (0.0%)	0.011^ a^
	155-165 cm	10 (55.6%)	8 (44.4%)	
	166-180 cm	34 (56.7%)	26 (43.3%)	
	>180 cm	2 (40.0%)	3 (60.0%)	
Weight	50-65 kg	10 (58.8%)	7 (41.2%)	0.249^ a^
	66-80 kg	11 (61.1%)	7 (38.9%)	
	81-95 kg	12 (44.4%)	15 (55.6%)	
	>95 kg	12 (60.0%)	8 (40.0%)	
Suffer from arthritis?	No	31 (54.4%)	26 (45.6%)	0.324^ a^
	Yes	16 (61.5%)	10 (38.5%)	
Location of injury	Right knee	18 (54.5%)	15 (45.5%)	0. 230^ a^
	Left knee	20 (80.0%)	5 (20.0%)	
	Both knees	2 (50.0%)	2 (50.0%)	
Time from injury to surgery?	<1 month	4 (44.4%)	5 (55.6%)	0.304^ a^
	1-6 months	7 (77.8%)	2 (22.2%)	
	6-12 months	8 (66.7%)	4 (33.3%)	
	>12 months	18 (60.0%)	12 (40.0%)	
Other knee injuries?	No	34 (52.3%)	31 (47.7%)	1.000^a^
	Yes	10 (66.7%)	5 (33.3%)	
Functional limitations of the knee after injury	Severe limitations/dysfunction	19 (95.0%)	1 (5.0%)	<0.001^ a^
	Moderate to severe functional limitations	28 (100.0%)	0 (0.0%)	
	Mild to moderate functional limitations	0 (0.0%)	30 (100.0%)	
	Normal or near-normal function	0 (0.0%)	6 (100.0%)	
During the past four weeks or since the onset of pain, how much pain have you felt?	Mean (SD)	4.35 ± 3.19	1.77 ± 1.54	0.005^ b^
If you feel pain, how severe is it?	Mean (SD)	4.06 ± 2.82	2.08 ± 1.61	0.016^ b^
Knee performance before injury	Mean (SD)	4.99 ± 4.09	7.08 ± 4.57	0.101^ b^
Knee performance in the current condition	Mean (SD)	4.20 ± 3.15	5.85 ± 3.44	0.094^ b^
TSK-11 score	Mean (SD)	22.90 ± 3.49	15.77 ± 2.05	<0.001^ b^
IKDC score	Mean (SD)	63.02 ± 17.64	81.01 ± 9.47	<0.001^ b^

## Discussion

The ACL is the key stabilizer of the knee, which prevents the anterior translation and the excessive rotation of the knee joint. Injuries to this ligament are very common in sports, often occurring through non-contact mechanisms like sudden directional changes [6]. The management of this condition may be surgical or conservative, which is dependent on the activity level and the risk of reinjury. ACLR is the gold standard for active individuals who aim to restore the pre-injury function and want to return to their sport [22]. Despite the successful surgery, many patients do not RTS, which could be due to psychological factors like kinesiophobia and pain in their knee [23]. This study aimed to explore the factors that influence the RTS in the Saudi population.

This study also highlights one of the most prominent findings, which is the influence of psychological fear, specifically kinesiophobia, on RTS decisions. Based on the TSK-11 score, 79.8% of participants experienced moderate fear, and only 15.5% demonstrated minimal fear. This aligns with previous studies, such as those by Hsu et al. (2017), which emphasized the substantial role of fear of re-injury as a psychological hurdle for returning to pre-injury sports [24].

Furthermore, there is a significant inverse relationship between TSK-11 and IKDC scores (r = -0.410, p < 0.001), confirming that higher fear correlates with poorer knee function and lower RTS rates. This supports the biopsychosocial model highlighted by Piussi et al. (2025), which stresses the need for integrated care involving both physical and psychological rehabilitation strategies [25].

Interestingly, no female participants returned to sport, although this finding was not statistically significant due to the small female sample size (n=9). Previous literature has reported lower RTS rates among females, citing factors such as lower muscle strength recovery, societal expectations, and greater fear of reinjury (Klemm et al., 2023) [26]. Our findings underscore the need for gender-sensitive rehabilitation strategies to ensure better reintegration into sport for female athletes.

Notably, there is a strong association between pain levels and RTS. Participants who returned to sport reported significantly lower pain scores (mean 1.77 vs. 4.35, p = 0.005) and less perceived pain severity (mean 2.08 vs. 4.06, p = 0.016). This supports the assertion by Betsch et al. (2021) that pain modulation is a crucial determinant of readiness to return [27]. While over half of the participants reported no swelling or stiffness, a substantial proportion (33.3% and 41.7%, respectively) experienced these symptoms, suggesting unresolved inflammation or poor muscular control may persist long after surgery.

Moreover, the functional tasks involving dynamic control, such as squatting (36.6% very difficult), jumping (17.1% unable), and kneeling (15.9% very difficult), remained challenging for a considerable number of participants. These observations echo findings from Stasi et al. (2013), which stressed the importance of neuromuscular re-education and proprioceptive training in regaining complex movement confidence [28].

Regarding the demographic factors of RTS, height is significantly associated with better outcomes. Participants taller than 180 cm had higher RTS rates, which could reflect greater lower limb power or confidence in biomechanics, although this finding has limited support in the current literature and warrants further investigation. Time from injury to surgery did not reach statistical significance but revealed an important clinical observation that early surgery (<1 month) had the highest RTS rate (55.6%). Delayed intervention has previously been associated with worse outcomes and increased risk of secondary injuries (Reijman et al., 2021) [29]. Future studies may be needed to stratify the outcomes based on surgical timing more specifically.

Clinical implications

RTS after ACLR is not only influenced by physical recovery but also by psychological readiness. Persistent fear and functional limitations suggest a need for improved postoperative care. Clinical protocols should integrate routine kinesiophobia screening (e.g., TSK-11 scale), cognitive-behavioral strategies to address fear-avoidance, and tailored strength and proprioception training. Educating patients on gradual, safe activity resumption can enhance confidence, reduce fear, and ultimately improve the likelihood of returning to pre-injury sport levels.

Strengths and limitations

To the best of our knowledge, only one study has been conducted in Saudi Arabia examining factors affecting RTS. [17] The study was conducted only in one tertiary center. Factors specific to this center could skew the results. ACL injury is one of the most frequent injuries in the field of sports medicine. Knowing the drawbacks of returning to pre-injury-level sport would help athletes stand up after their injury. Furthermore, this study involved a larger female participation (10%) compared to the previous studies. Female participation in sports in Saudi Arabia has increased in recent years. In future studies, female representation would increase greatly.

This study has several limitations. The sample size was relatively small and predominantly male, limiting generalizability across genders. The cross-sectional design restricts the ability to establish causal relationships between fear, function, and RTS. Self-reported measures may introduce bias, and psychological variables were not assessed longitudinally. Future research should involve larger, more diverse populations, including female athletes, and adopt prospective designs to monitor recovery trajectories over time. The recurrence of this limitation in our study is attributed to the already low number of ACLR cases in the hospitals we retrospectively reviewed. Additionally, intervention-based studies targeting fear and functional deficits could offer valuable insights into improving RTS outcomes post ACLR.

## Conclusions

This study shows that returning to sport following ACLR is influenced by both physical and psychological factors. While less than half of the participants returned to their pre-injury activity level, those with better knee function, lower pain severity, and minimal kinesiophobia were significantly more likely to achieve RTS. Functional limitations, psychological fear, and persistent symptoms remain major barriers. These findings emphasize the importance of addressing both biomechanical recovery and psychological readiness through structured rehabilitation, pain management, and targeted psychological support to optimize post-ACLR outcomes.

## Figures and Tables

**Figure 3:**
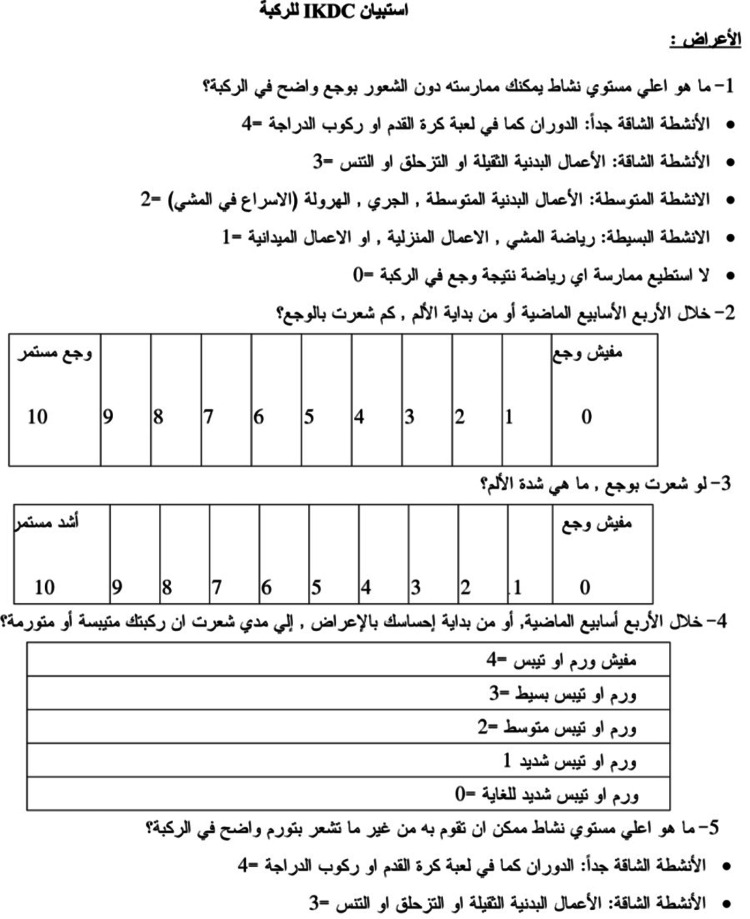
Arabic validated version of IKDC score (part 1) IKDC: International Knee Documentation Committee

**Figure 4:**
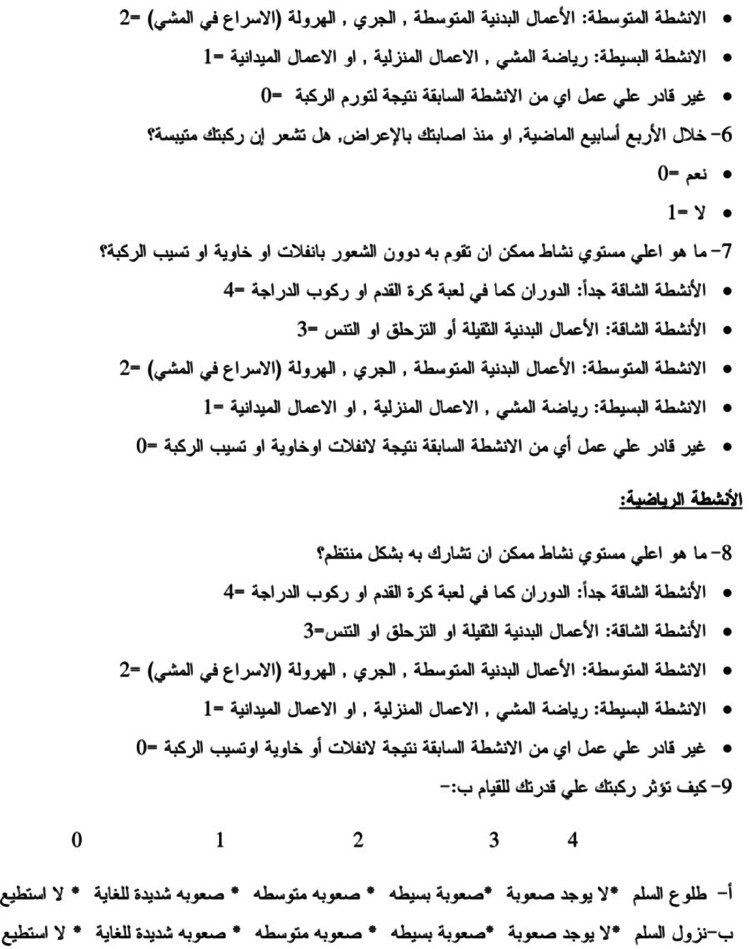
Arabic validated version of the IKDC score (part 2) IKDC: International Knee Documentation Committee

**Figure 5:**
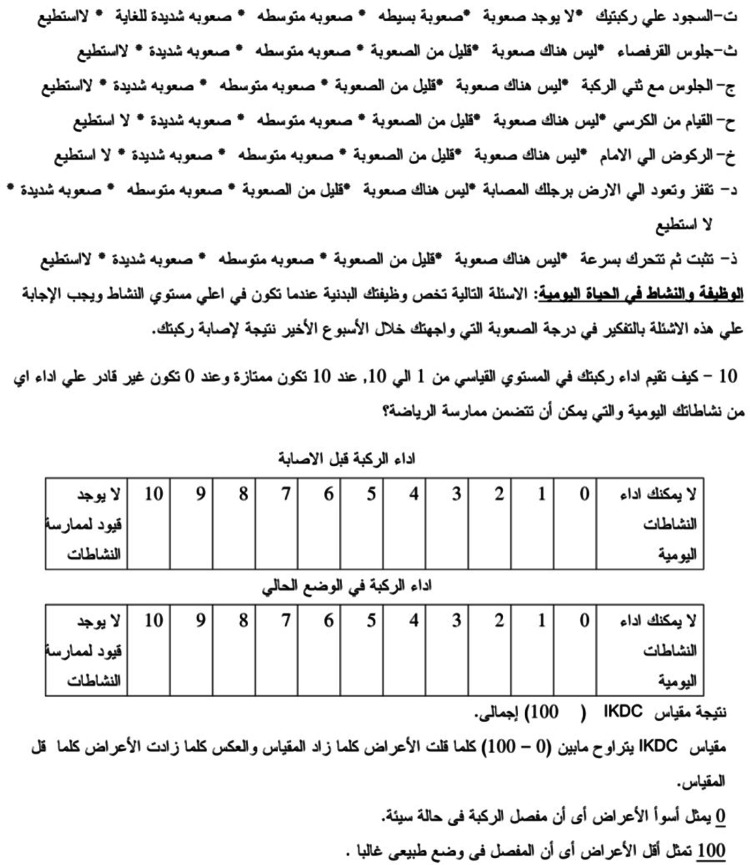
Arabic validated version of the IKDC score (part 3) IKDC: International Knee Documentation Committee

## Appendices

Appendix A 

**Table 6:** Validated, shortened version of the TSK-11 used in the study questionnaire TSK-11: Tampa Scale for Kinesiophobia

Tampa Scale for Kinesiophobia(TSK-11)	
I am afraid that I might injure myself if I exercise.	Strongly agree, Agree, Disagree, Strongly disagree
If I try to overcome it, my pain would increase.	Strongly agree, Agree, Disagree, Strongly disagree
Pain always means that I have injured my body.	Strongly agree, Agree, Disagree, Strongly disagree
I am afraid that I might injure myself accidentally.	Strongly agree, Agree, Disagree, Strongly disagree
It is really not safe for a person with a condition like mine to be physically active.	Strongly agree, Agree, Disagree, Strongly disagree
I can’t do all the things that normal people do because it is too easy for me to get injured.	Strongly agree, Agree, Disagree, Strongly disagree
No one should have to exercise when he/she is in pain.	Strongly agree, Agree, Disagree, Strongly disagree
My body is telling me I have something dangerously wrong.	Strongly agree, Agree, Disagree, Strongly disagree
My accident has put my body at risk for the rest of my life.	Strongly agree, Agree, Disagree, Strongly disagree
I wouldn’t have this much pain if there weren’t something potentially dangerous going on in my body.	Strongly agree, Agree, Disagree, Strongly disagree
Pain lets me know when to stop exercising so that I don’t injure myself.	Strongly agree, Agree, Disagree, Strongly disagree

Appendix B 

**Table 7:** Questionnaire used to collect demographic data for the study participants.

Demographic data	
What is your age?	Less than 18 18-30 30-40 More than 40
What is your gender?	Female Male
Are you married or single?	I am Married I am single
Are you an employee?	Yes, I am No, I am not
Where are you live?	Eastern / western / North / East
What is your height?	(….) cm
What is your weight?	(….) kg
Do you have osteoarthritis?	Yes No
Where is the site of the injury?	Right knee Left knee
The time between the injury and the surgery?	Less than 1month 1-6 months 6-12 months More than 1 year
Are there any other injuries?	Yes No

Appendix C 
